# Simpson’s Paradox is suppression, but Lord’s Paradox is neither: clarification of and correction to Tu, Gunnell, and Gilthorpe (2008)

**DOI:** 10.1186/s12982-019-0087-0

**Published:** 2019-11-27

**Authors:** Carol A. Nickerson, Nicholas J. L. Brown

**Affiliations:** 1 Champaign, IL USA; 2 Department of Health Sciences, University Medical Center, University of Groningen, 9713 GZ Groningen, The Netherlands

**Keywords:** Confounding, Contingency table, Epidemiology, Lord’s Paradox, Regression, Reversal paradox, Simpson’s Paradox, Suppression

## Abstract

Tu et al. (Emerg Themes Epidemiol 5:2, 2008. https://doi.org/10.1186/1742-7622-5-2) asserted that suppression, Simpson’s Paradox, and Lord’s Paradox are all the same phenomenon—the reversal paradox. In the reversal paradox, the association between an outcome variable and an explanatory (predictor) variable is reversed when another explanatory variable is added to the analysis. More specifically, Tu et al. (2008) purported to demonstrate that these three paradoxes are different manifestations of the same phenomenon, differently named depending on the scaling of the outcome variable, the explanatory variable, and the third variable. According to Tu et al. (2008), when all three variables are continuous, the phenomenon is called suppression; when all three variables are categorical, the phenomenon is called Simpson’s Paradox; and when the outcome variable and the third variable are continuous but the explanatory variable is categorical, the phenomenon is called Lord’s Paradox. We show that (a) the strong form of Simpson’s Paradox is equivalent to negative suppression for a ${2 \times 2 \times 2}$ contingency table, (b) the weak form of Simpson’s Paradox is equivalent to classical suppression for a ${2 \times 2 \times 2}$ contingency table, and (c) Lord’s Paradox is not the same phenomenon as suppression or Simpson’s Paradox.

Tu, Gunnell, and Gilthorpe’s *Emerging Themes in Epidemiology* article [1] asserted that suppression, Simpson’s Paradox, and Lord’s Paradox are all the same phenomenon—the reversal paradox. In the reversal paradox, the association between an outcome variable and an explanatory (predictor) variable is reversed (changes sign) when another explanatory variable (which may be called a third variable, a covariate, a confounding variable, a disturber, a concomitant variable, a control variable, a background variable, or a lurking variable) is added to the analysis [2]. More specifically, Tu et al. [1] purported to demonstrate that these three paradoxes are different manifestations of the same phenomenon, differently named depending on the scaling of the outcome variable, the explanatory variable, and the third variable. According to Tu et al. [1], when all three variables are continuous, the phenomenon is called suppression; when all three variables are categorical; the phenomenon is called Simpson’s Paradox; and when the outcome variable and the third variable are continuous but the explanatory variable is categorical, the phenomenon is called Lord’s Paradox.

Tu et al. [1] are partly right and partly wrong. The inaccuracies in their presentation stem from their

- failing to distinguish between the three different types of suppression, only one of which involves an association reversal;
- failing to distinguish between the strong form of Simpson’s Paradox, in which there is an association reversal, and the original weak form, in which there is not [3]; and
- misunderstanding Lord’s Paradox, which cannot be equated with either Simpson’s Paradox or suppression.^1^

Because Tu et al.’s [1] article seems to have had considerable influence in the decade or so since it was published—Google Scholar indicates that it has been cited more than 180 times—it is important that these inaccuracies be rectified. We clarify and correct Tu et al.’s [1] presentation by first describing suppression, Simpson’s Paradox, and Lord’s Paradox. We next employ Cornfield’s inequality [4] to show mathematically that suppression and Simpson’s Paradox are indeed the same phenomenon, as Tu et al. [1] asserted, and provide a simple example of Lord’s Paradox to demonstrate that it is neither suppression nor Simpson’s Paradox, contrary to Tu et al.’s [1] claim. We then examine Tu et al.’s [1] hypothetical examples of suppression, Simpson’s Paradox, and Lord’s Paradox. We conclude briefly by agreeing with Tu et al. [1] that these paradoxes have serious implications for the interpretation of evidence from observational studies that employ contingency-table analyses or regression-based models but emphasize the importance of accurate descriptions of these paradoxes and the relations between them.

## Suppression

Consider an ordinary least squares regression with a criterion or outcome variable *Y* and two possible explanatory or predictor variables ${X_1}$ and ${X_2}$. For simplicity and with no loss of generality, we assume that all three variables are standardized, and that the variables are scored so that the correlation between the two explanatory variables (${r_{X_1X_2}}$) is greater than or equal to zero and the correlation between the outcome variable and the first explanatory variable (${r_{YX_1}}$) is positive.^2^ Then the correlation between the outcome variable and the second explanatory variable (${r_{YX_2}}$) may be negative, zero, or positive. The simple regression coefficient for ${X_1}$ or ${X_2}$ is obtained by regressing *Y* on either ${X_1}$ alone or ${X_2}$ alone, respectively. With standardized variables, the simple regression coefficient is equivalent to the correlation between the explanatory variable and the outcome variable. The partial regression coefficient (${\beta }$) for ${X_1}$ and for ${X_2}$ is obtained by regressing *Y* on both ${X_1}$ and ${X_2}$. These partial regression coefficients can also be computed from the three correlations as follows:

$$\begin{aligned} {\beta _1} &= (r_{YX_1} - r_{YX_2} \times r_{{X_1}{X_2}}) / (1 - {r_{{X_1}{X_2}}}^2)\\ {\beta _2}& = (r_{YX_2} - r_{YX_1} \times r_{{X_1}{X_2}}) / (1 - {r_{{X_1}{X_2}}}^2). \end{aligned}$$

Researchers often seem to believe that, in a regression predicting an outcome variable from two explanatory variables, one or the other of two situations must occur: either ${X_1}$ and ${X_2}$ are *independent*, or ${X_1}$ and ${X_2}$ are *redundant*:

- *Independence* occurs when the two explanatory variables are uncorrelated. The partial regression coefficient for each of the two explanatory variables then equals its corresponding simple regression coefficient. For example, if the correlations between the three variables *Y*, ${X_1}$, and ${X_2}$—${r_{YX_1}}$, ${r_{YX_2}}$, and ${r_{X_1X_2}}$—equal .44, .33, and .00, respectively, the partial regression coefficients for ${X_1}$ and ${X_2}$ equal .44 and .33.
- *Redundancy* occurs when the two explanatory variables are correlated. Each partial regression coefficient has the same sign as, but is less than, its corresponding simple regression coefficient. For example, if the correlations ${r_{YX_1}}$, ${r_{YX_2}}$, and ${r_{X_1X_2}}$ equal .44, .33, and .60, respectively, the partial regression coefficients for ${X_1}$ and ${X_2}$ equal .38 and .10 [5, Figure 1, p. 308]. Redundancy is the most common regression situation.

But three other situations are possible when the two explanatory variables are correlated: *reciprocal suppression*, *classical suppression*, and *negative suppression* [6, pp. 84–91, 7–9].

- *Reciprocal suppression* (also called *cooperative suppression*) occurs whenever the correlation between the outcome variable and the second explanatory variable is negative. The partial regression coefficient for each of the two explanatory variables is greater than its corresponding simple regression coefficient but the sign is unchanged. For example, if the correlations ${r_{YX_1}}$, ${r_{YX_2}}$, and ${r_{X_1X_2}}$ equal .44, ${-\,.20}$, and .60, respectively, the partial regression coefficients for ${X_1}$ and ${X_2}$ equal .88 and ${-\,.73}$ [5, Figure 1, p. 308]. Because reciprocal suppression does not involve a sign reversal, its occurrence cannot be considered an example of the reversal paradox.
- *Classical suppression* (also called *traditional suppression*) occurs whenever the correlation between the outcome variable and the second explanatory variable equals zero (or in some presentations, nearly zero). The partial regression coefficient for the explanatory variable having the zero correlation with the outcome variable is negative; the partial regression coefficient for the explanatory variable having the non-zero correlation with the outcome variable has the same sign as, but is greater than, its corresponding simple regression coefficient. For example, if the correlations ${r_{YX_1}}$, ${r_{YX_2}}$, and ${r_{X_1X_2}}$ equal .44, .00, and .60, respectively, the partial regression coefficients for ${X_1}$ and ${X_2}$ equal .69 and ${-\,.41}$ [5, Figure 1, p. 308]. Because classical suppression involves a change from a zero association between one of the explanatory variables and the outcome variable to a non-zero association, its occurrence, strictly speaking, cannot be considered an example of the reversal paradox.

Reciprocal suppression always occurs when the correlation between the outcome variable and the second explanatory variable is negative, and classical suppression always occurs when the correlation between the outcome variable and the second explanatory variable is zero (assuming that the correlation between the outcome variable and the first explanatory variable is positive, and that the correlation between the two explanatory variables is positive, as explained earlier).

- *Negative suppression* (also called *net suppression*) can, but does not necessarily, occur when the correlation between the outcome variable and the second explanatory variable is positive. Negative suppression occurs whenever the correlation between the two explanatory variables (${r_{X_1X_2}}$) is greater than the ratio of the correlations of the two explanatory variables with the outcome variable, with the smaller of these two correlations placed in the numerator of the ratio and the larger placed in the denominator, so that the ratio is less than or equal to 1.00. That is, negative suppression occurs if
  $$\begin{aligned} \begin{array}{ccc} r_{X_1 X_2}> r_{Y X_1} / r_{Y X_2} & & \text{if}\ \,r_ {Y X_2}> r_{Y X_1} \\ \\ & \text{or} & \\ \\ r_{X_1 X_2}> r_{Y X_2} / r_{Y X_1} & & \text{if}\ \,r_ {Y X_1} > r_{Y X_2} .\\ \end{array} \end{aligned}$$
  Otherwise, redundancy occurs. In negative suppression, the partial regression coefficient for the explanatory variable that has the larger correlation with the outcome variable keeps the same sign and is greater than its corresponding simple regression coefficient. The partial regression coefficient for the explanatory variable that has the smaller correlation with the outcome variable reverses sign and can be less than, equal to, or greater in magnitude than its corresponding simple regression coefficient. For example, if the correlations ${r_{YX_1}}$, ${r_{YX_2}}$, and ${r_{X_1X_2}}$ equal .44, .10, and .60, respectively, negative suppression occurs because .60 is greater than ${.10/.44 = .23}$. The partial regression coefficients for ${X_1}$ and ${X_2}$ equal .59 and ${-\,.26}$, respectively [5, Figure 1, p. 308; see also 10]. The occurrence of negative suppression is an example of the reversal paradox.

Although the occurrence of classical suppression *is not* an example of the reversal paradox (because there is no sign reversal), and the occurrence of negative suppression *is* an example of the reversal paradox (because there is a sign reversal), classical suppression can be regarded nonethless as a special case of negative suppression because, whenever the correlation of one of the explanatory variables with the outcome variable equals zero, the correlation between the two explanatory variables (which is positive) must exceed the ratio of the correlations of the two explanatory variables with the outcome variable (which equals zero).

Suppression can occur in regression models with more than two explanatory variables, but its operation is more complicated [7, 11] and has not been much investigated in the statistical literature. Tu et al. [1] focused on the case of two explanatory variables, so we will not consider further here the case of more than two.

## Simpson’s Paradox

Simpson [12] noted that in a ${2 \times 2 \times 2}$ contingency table, with the level of each of the three dichotomous variables coded 0 or 1, there can be an association of two of the three variables at each level of the third variable although there is no overall association of the two variables. He provided a hypothetical medical example with an outcome variable “status” (alive, dead), an explanatory variable “treatment” (untreated, treated), and a third variable “sex” (male, female), with the table cell frequencies shown in Table 1. When sex is disregarded, there is no association between treatment and status; the untreated and the treated persons have the same probability of death (.50). When sex is considered, there is a negative association between treatment and status for both males and females, with untreated males having a higher probability of death than treated males (.43 vs. .38), and untreated females having a higher probability of death than treated females (.60 vs. .56).^3^

**Table 1:** Associations between treatment and status. Adapted from Simpson [11, Item 10, p. 241]

	Untreated	Treated			
Association between treatment and status, disregarding sex					
Alive	6	20			
Dead	6	20			
Total	12	40			
Probability dead	.50	.50	No association		

	Male		Female		
	Untreated	Treated	Untreated	Treated	
Association between treatment and status for each sex					
Alive	4	8	2	12	
Dead	3	5	3	15	
Total	7	13	5	27	
Probability dead	.43	.38	.60	.56	Negative association for both male and female

Status coded 0 = alive, 1 = dead; treatment coded 0 = untreated, 1 = treated; sex coded 0 = male, 1 = female

Simpson [12] described the weak form of the paradox that now bears his name, although the phenomenon was known much earlier [13, 14; see also 3]. In the weak form of Simpson’s Paradox, a lack of association between two variables transmutes into a positive or negative association when a third variable is considered. Strictly speaking, the occurrence of the weak form of Simpson’s Paradox, like classical suppression, cannot be considered an example of the reversal paradox. Since 1951, when Simpson wrote his article, Simpson’s Paradox usually has been defined and/or demonstrated in terms of an actual association reversal, which is the strong form of Simpson’s Paradox. For example, Charig, Webb, Payne, and Wickham [15] showed that the association between the “surgical outcome” (failure, success) and the “type of surgery” (open surgery, percutaneous nephrolithotomy) for kidney stones reversed when the “kidney-stone size” (large, small) was taken into account. Charig et [15] presented their results in terms of percentages rather than probabilities. As shown in Table 2, when kidney-stone size was disregarded, ${83\%}$ of the percutaneous nephrolithotomies were successful, whereas only ${78\%}$ of the open surgeries were successful. But when surgical outcome was examined separately for large kidney stones and small kidney stones, open surgeries were more successful than percutaneous nephrolithotomies for both large kidney stones (73 vs. ${69\%}$) and small kidney stones (93 vs. ${87\%}$).^4^ Simpson’s Paradox has also been observed in contingency tables larger than ${2 \times 2 \times 2}$ (usually ${2 \times 2 \times k}$). For example, Simpson’s Paradox occurred in a contingency table with two airlines (America West, Alaska), two performances (on time, delayed), and five cities (Los Angeles, Phoenix, San Diego, San Francisco, Seattle). Although America West Airlines had a higher percentage of on-time flights overall than did Alaska Airlines, Alaska Airlines had a higher percentage of on-time flights for each of the five cities [16].

**Table 2:** Associations between type of surgery and surgical outcome. Adapted from Charig et al. [14, Tables I and II, p. 880]

	Open surgery	Percutaneous nephrolithotomy			
Association between type of surgery and surgical outcome, disregarding kidney-stone size					
Failure	77	61			
Success	273	289			
Total	350	350			
Percentage success	78%	83%	Positive association		

	Large stone		Small stone		
	Open surgery	Percutaneous nephrolithotomy	Open surgery	Percutaneous nephrolithotomy	
Association between type of surgery and surgical outcome for each kidney-stone size					
Failure	71	25	6	36	
Success	192	55	81	234	
Total	263	80	87	270	
Percentage success	73%	69%	93%	87%	Negative associations

Surgical outcome coded 0 = failure, 1 = success; type of surgery coded 0 = open surgery, 1 = percutaneous nephrolithotomy; kidney-stone size coded 0 = large stone, 1 = small stone

## Lord’s Paradox

Lord [17] described a problem in the interpretation of studies examining the relation between an outcome variable and a pre-existing group variable when both a pretest measure and a posttest measure of the outcome variable are available. Suppose that a university is interested in determining whether the diet provided in its dining halls has an effect on the weight of the students, and whether there might be a sex difference in this effect. Student weight is assessed twice, at the beginning of the school year in September, and at the end of the school year in June. Lord [17] noted that two different ways of analyzing the data yield different results. When the outcome variable (June or posttest weight) is regressed on both the group variable (the explanatory variable sex) and the third (or control) variable (September or pretest weight), there is a significant effect of sex on June weight, with men being heavier. When the difference between the June weight and the September weight is regressed on sex, however, there is no significant effect of sex.

As was the case with the (weak) form of Simpson’s paradox presented by Simpson [12], Lord’s Paradox, as presented by Lord [17], did not describe an association reversal, but a change from no association to an association (here, assuming that sex is coded 0 for women and 1 for men, a positive association). We are unaware of published examples of Lord’s Paradox in which there is an actual association reversal, but certainly it is the case that an association reversal can occur when the same data set is analyzed using these two different methods of analysis.

## Simpson’s Paradox is suppression

Suppression focuses on the changes to the regression coefficients when a second explanatory variable is added to a regression model containing only one explanatory variable. That is, an examination of suppression compares the signs and magnitudes of the regression coefficients (alternatively, part correlations or partial correlations [9]) for the regressions predicting *Y* from ${X_1}$ alone and *Y* from ${X_2}$ alone to the signs and the magnitudes of the regression coefficients for the regression predicting *Y* from both ${X_1}$ and ${X_2}$. Simpson’s Paradox focuses on changes to probabilities, ratios, or percentages computed for a ${2 \times 2}$ contingency table to the probabilities, ratios, or percentages computed for the two subtables of a ${2 \times 2 \times 2}$ contingency table created by considering a third variable. Nonetheless, the strong form of Simpson’s Paradox is equivalent to negative suppression and the weak form of Simpson’s Paradox is equivalent to classical suppression, as consideration of “Cornfield’s inequality” [4] shows.

Cornfield et al. [4; see also 18] derived the necessary conditions for a third (“common cause”) variable to account for the observed association between an explanatory (“apparent cause”) variable and an outcome variable, assuming that this observed association is spurious. These conditions establish the minimum effect size necessary for the third variable to reverse the observed association, resulting in Simpson’s Paradox. For simplicity, we use the following mnemonic notation: *O* and ${O'}$ represent the 1 and 0 values of the outcome, *A* and ${A'}$ represent the 1 and 0 values of the apparent cause, and *C* and ${C'}$ represent the 1 and 0 values of the common cause in a ${2 \times 2 \times 2}$ contingency table. *P*(*O*) is the probability of *O*, *P*(*O*|*A*) is the probability of *O* given that *A* has occurred, and so on. *P*(*O*) and ${P(O')}$ sum to 1, of course, and analogously for the other two variables. Cornfield et al. [4] explicitly assumed the association between the outcome (*O*) and the common cause (*C*), and the association between the apparent cause (*A*) and the common cause (*C*), to be positive, which seems reasonable in a disease context. One way of expressing Cornfield’s inequality is

$$\begin{aligned} {P(O|A) - P(O|A') = [P(O|C) - P(O|C')] \times [P(C|A) - P(C|A')]}. \end{aligned}$$

Because ${P(C|A) - P(C|A')}$ is less than or equal to 1,

$$\begin{aligned} {P(O|C) - P(O|C') \ge P(O|A) - P(O|A')}. \end{aligned}$$

That is, to reverse the observed association between the outcome and the apparent cause, the association between the outcome and the common cause must be stronger than the association between the outcome and the apparent cause.

Cornfield’s inequality can also be expressed in terms of correlations. When the variables are dichotomous, the correlation *r* is equivalent to the ${\phi }$ coefficient, a measure of association for contingency tables. The ${\phi }$ coefficient for a ${2 \times 2}$ contingency table can be expressed in terms of probabilities. For example, for the variables *O* and *C*,

$$\begin{aligned} {\phi _{OC} = [P(O|C) - P(O|C')]} \times {\sqrt{[P(C) \times P(C')] / [P(O) \times P(O')]}} \end{aligned}$$

and analogously for the variable pairs *O* and *A*, and *A* and *C*.

If the association between the outcome *O* and the apparent cause *A* is completely due to the association between each of these two variables and the common cause *C*, then

$$\begin{aligned} {\phi _{OA} = \phi _{OC} \times \phi _{AC}} \end{aligned}$$

or, in terms of *r*,

$$\begin{aligned} {r_{OA} = r_{OC} \times r_{AC}}. \end{aligned}$$

Rearranging terms gives

$$\begin{aligned} {r_{AC} = r_{OA} / r_{OC}}. \end{aligned}$$

Substituting *Y* for *O*, ${X_1}$ for *A*, and ${X_2}$ for *C* shows that this is the boundary for negative suppression described earlier for continuous variables:

$$\begin{aligned} {{r_{X_1X_2}} = {r_{YX_1}} / {r_{YX_2}}}. \end{aligned}$$

Thus, the strong form of Simpson’s Paradox is equivalent to suppression—specifically, negative suppression—for a ${2 \times 2 \times 2}$ contingency table, as Tu et al. [1] asserted.

To make all this concrete, consider again the kidney-stone example of the strong form of Simpson’s Paradox in Table 2. Table 3 rearranges the data in the bottom panel of Table 2 into three ${2 \times 2}$ contingency tables—one for surgical outcome by type of surgery, one for surgical outcome by kidney-stone size, and one for type of surgery by kidney-stone size—and includes all the relevant probabilities and ${\phi }$ coefficients. Cornfield’s inequality indicates that an association reversal will occur between the outcome variable (surgical outcome) and the treatment variable (apparent cause: type of surgery) when the third or confounding variable (common cause: kidney-stone size) is considered because the association between the outcome variable and the third variable [${P(O|C) {-} P(O|C')}$; ${.88 {-} .72 = .16}$] is greater than the association between the outcome variable and the treatment variable [${P(O|A) {-} P(O|A')}$; ${.83 {-} .78 = .05}$]. Analogously, comparison of the ${\phi }$ coefficients for the three ${2 \times 2}$ tables shows that negative suppression, and thus an association reversal, must occur when the third variable is added to the regression predicting the outcome variable from the treatment variable. The ${\phi }$ coefficients for the three contingency tables in Table 3 equal .06, .20, and .52, respectively. The ${\phi }$ coefficient for the treatment variable and the third variable (.52) is greater than the ratio of the ${\phi }$ coefficients of each of these variables with the outcome variable (${.06/.20 = .30}$), indicating that negative suppression must occur and that the regression coefficient for the treatment variable (which has the smaller association with the outcome variable) will reverse sign. The regression coefficient for the treatment variable changes from .06 to ${-\,.23}$, indicating a positive association between type of surgery (with open surgery coded 0 and percutaneous nephrolithotomy coded 1) when kidney-stone size is not included in the regression but a negative association when it is.

**Table 3:** Three $2 \times 2$ contingency tables for the data in Table 2

	Open surgery	Percutaneous nephrolithotomy		
Association between type of surgery and surgical outcome, disregarding kidney-stone size				
Failure	77	61		
Success	273	289		
Total	350	350		
Percentage success	78%	83%	Difference = 5%	$\phi = .06$

	Large stone	Small stone		
Association between kidney-stone size and surgical outcome, disregarding type of surgery				
Failure	96	42		
Success	247	315		
Total	343	357		
Percentage success	72%	88%	Difference = 16%	$\phi = .20$

	Open surgery	Percutaneous nephrolithotomy		
Association between type of surgery and kidney-stone size, disregarding surgical outcome				
Large stone	263	80		
Small stone	87	270		
Total	350	350		
Percentage small stone	25%	77%	Difference = 52%	$\phi = .52$

Surgical outcome coded 0 = failure, 1 = success; type of surgery coded 0 = open surgery, 1 = percutaneous nepholithotomy; kidney-stone size coded 0 = large stone, 1 = small stone

Although Cornfield et al. [4] did not explicitly mention the situation where the association between the outcome and the apparent cause is null—the weak form of Simpson’s Paradox—Cornfield’s inequality applies here as well, because ${P(O|A) -P(O|A') = 0}$ and hence is less than ${P(O|C) -P(O|C')}$, which is positive, and so the association between the outcome and the apparent cause at each level of the common cause will be non-null. Thus, the weak form of Simpson’s Paradox is equivalent to classical suppression for a ${2 \times 2 \times 2}$ contingency table.

For Simpson’s [12] example, Table 4 rearranges the data in the bottom panel of Table 1 into three ${2 \times 2}$ contingency tables—one for death by treatment, one for death by sex, and one for treatment by sex—and includes all the relevant probabilities and ${\phi }$ coefficients. Cornfield’s inequality indicates that the null association between the outcome variable (death) and the treatment variable (apparent cause: treatment) will become non-null when the third or confounding variable (common cause: sex) is considered because the association between the outcome variable and the third variable [${P(O|C) {-} P(O|C')}$; ${.56 {-} .40 = .16}$] is greater than the association between the outcome variable and the treatment variable [${P(O|A) {-} P(O|A')}$; ${.50 {-} .50 = .00}$]. Analogously, comparison of the ${\phi }$ coefficients for the three ${2 \times 2}$ contingency tables shows that classical suppression, and thus a change from a null association to a non-null association, must occur when the third variable is added to the regression predicting the outcome variable from the treatment variable. The ${\phi }$ coefficients for the three contingency tables in Table 4 equal .00, .16, and .22, respectively. In regression, a correlation of zero between the outcome variable and either one of the two explanatory variables guarantees that classical suppression will occur. The regression coefficients for ${X_1}$ and ${X_2}$ equal .17 and ${-\,.04}$, indicating a small negative association between treatment and status when sex is considered.

**Table 4:** Three 2 *times* 2 contingency tables for the data in Table 1

	Untreated	Treated		
Association between treatment and status, disregarding sex				
Alive	6	20		
Dead	6	20		
Total	12	40		
Probability dead	.50	.50	Difference = 0	$\phi = .00$

	Male	Female		
Association between sex and status, disregarding treatment				
Alive	12	14		
Dead	8	18		
Total	20	32		
Probability dead	.40	.56	Difference = .16	$\phi = .16$
Association between sex and treatment, disregarding status				
Untreated	7	5		
Treated	13	27		
Total	20	32		
Probability treated	.65	.84	Difference = .19	$\phi = .22$

Status coded 0 = alive, 1 = dead; treatment coded 0 = untreated, 1 = treated; sex coded 0 = male, 1 = female

**Table 5:** Hypothetical data for Lord’s Paradox

Group	Pretest	Posttest
0	10	20
0	20	25
0	30	30
0	40	35
0	50	40
1	50	60
1	60	65
1	70	70
1	80	75
1	90	80

## Lord’s Paradox is not suppression or Simpson’s Paradox

Contrary to Tu et al.’s [1] claim, Lord’s Paradox cannot be equated with any type of suppression or with either the weak or the strong form of Simpson’s Paradox. All three forms of suppression depend on a comparison of the regression coefficients for ${X_1}$ and ${X_2}$ between the regressions with one explanatory variable

$$\begin{aligned}&Y \leftarrow {X_1}\\&Y \leftarrow {X_2}\\ \end{aligned}$$

and the regression with two explanatory variables

$$\begin{aligned} Y \leftarrow {X_1}\,{X_2}. \end{aligned}$$

Lord’s Paradox, on the other hand, refers to a comparison of the regression coefficients for ${X_1}$ between

$$\begin{aligned} (Y - {X2}) \leftarrow {X_1} \end{aligned}$$

and

$$\begin{aligned} Y \leftarrow {X_1}\,{X_2}. \end{aligned}$$

The first regression is based on a difference-score definition of change, whereas the second regression is based on a residual-score definition of change. A difference score is computed by subtracting the pretest value from the posttest value and has a straightforward interpretation. A positive difference score means that the score has increased from the pretest to the posttest; a negative difference score means that the score has decreased from the pretest to the posttest. A residual score is computed by regressing the posttest on the pretest, or by including both the pretest and the posttest as explanatory variables. A residual score indicates whether the posttest score has changed more or less than expected based on the pretest score and the regression. A positive residual score means that the posttest score is larger than expected; a negative residual score means that the posttest score is smaller than expected. Lord’s Paradox is not actually a paradox, then, because the results of analyses based on two different definitions of change are not comparable. The two different analyses do not ask the same question of the data.

The apparent distinction between the regression based on difference scores and the regression based on residual scores for Lord’s Paradox is that the latter has the pretest ${X_2}$ on the right side of the equation but the former does not. Rewriting the equations more formally

$$\begin{aligned} (Y - {X2}) = {\beta _1}{X_1} + e \end{aligned}$$

and

$$\begin{aligned} Y = {\beta _1}{X_1} +{\beta _2}{X_2} + e \end{aligned}$$

(*e* represents error) and then adding ${X_2}$ to both sides of the former

$$\begin{aligned} Y = {\beta _1}{X_1} + {X_2} + e \end{aligned}$$

shows that the actual distinction between the two regressions is that in the difference-score regression, the regression coefficient for the pretest ${X_2}$ is forced to equal 1. Put differently, for pretest-posttest data, the difference-score regression and the residual-score regression will give exactly the same results if and only if the slope of the within-group regression line predicting posttest from pretest equals 1 for each group.

A simple example shows that suppression is not necessary for Lord’s Paradox to occur. Consider the small hypothetical data set in Table 5.^5^ This data set exhibits Lord’s Paradox. Regressing posttest on group and pretest shows that there is no effect at all of group on posttest. Regressing the difference between posttest and pretest on group does show a significant effect of group, however. The correlations between group and pretest, group and posttest, and posttest and pretest equal .82, .94, and .96, respectively. The correlation of the two explanatory variables (.82) does not exceed the ratio of the correlation of each explanatory variable with the outcome variable (${.94 / .96 = .98}$) so there is no sign reversal and no negative suppression. Neither explanatory variable has a zero correlation with the outcome variable, so there is no classical suppression, and all three correlations are positive, so there is no reciprocal suppression. This example demonstrates that Lord’s Paradox is not the same as suppression and hence, not the same as Simpson’s Paradox. For reasons that are unclear, a few other authors have made the same mistake of considering Lord’s Paradox to be the same as Simpson’s Paradox (e.g., [19, 20]).

## Tu et al.’s [1] three examples

Tu et al. [1] presented a hypothetical example of suppression, of Simpson’s Paradox, and of Lord’s Paradox. The motivation for these three examples is the “fetal origins of adult disease” (FOAD) hypothesis developed by the epidemiologist Barker [21], which suggests a possible association between low birth weight and various chronic diseases in adulthood (e.g., hypertension, diabetes, coronary artery disease). The question is whether current (adult) weight should be considered in analyzing this association; many studies have found an inverse association between birth weight and adult disease only when current weight (or some other measure of adult body size) is considered. In Tu et al.’s [1] three hypothetical examples, the outcome variable is systolic blood pressure, the explanatory variable is birth weight, and the third variable is current weight.

### Example of suppression

For their example of suppression, Tu et al. [1] simulated continuous values of systolic blood pressure, birth weight, and current weight for 1000 adult men so that the correlation between each pair of three variables is positive.^6^ The correlation between blood pressure and birth weight equals .11,^7^ the correlation between blood pressure and current weight equals .50, and the correlation between birth weight and current weight equals .52. Note that the association between birth weight and blood pressure is positive and significant in these simulated data. When current weight is considered, however, the association between birth weight and blood pressure becomes negative and remains significant. As Tu et al. [1] noted, consideration of current weight reversed and increased the association between birth weight and blood pressure, suggesting that low birth weight leads to adult hypertension.

Tu et al. [1, p. 6] correctly stated that the analysis on these continuous variables is characterized by suppression, but mistakenly explained this example in terms of classical suppression, whereas in fact their example demonstrates negative suppression. The correlation between the explanatory variable (birth weight) and the third variable (current weight) is greater than the ratio of the correlation of each of these variables with the outcome variable (blood pressure): ${.52 > (.11/.50 = .22)}$. The regression coefficient for the third variable increases from .50 to .61; the regression coefficient for the explanatory variable reverses sign and increases in magnitude from .11 to ${-\,.21}$ and remains significant.

### Example of Simpson’s Paradox

For their example of Simpson’s Paradox, Tu et al. [1, Table 2, p. 3] dichotomized the three simulated continuous variables blood pressure (normal, high), birth weight (low, high), and current weight (low, high). They then first cross-classified blood pressure by birth weight, disregarding current weight, showing that the probability of developing high blood pressure is higher for persons with a high birth weight than it is for persons with a low birth weight (.362 vs. .272); that is, the association between birth weight and blood pressure is positive and significant in these simulated data. They then considered current weight by cross-classifying blood pressure by birth weight for each of the two values of current weight. When current weight is considered, the probability of developing high blood pressure is lower for persons with a high birth weight than it is for persons with a low birth weight, both for persons with a low current weight (.199 vs. .231) and for persons with a high current weight (.550 vs. .569). That is, when current weight is considered, the association between birth weight and blood pressure becomes negative. (Tu et al. [1] apparently did not realize that this negative association is not significant.) This association reversal exemplifies the strong version of Simpson’s Paradox. As in Tu et al.’s [1] example of suppression, in their example of Simpson’s Paradox, consideration of current weight reversed the association between birth weight and blood pressure, suggesting that low birth weight might lead to adult hypertension. This is not surprising, given that this example of Simpson’s Paradox is also an example of negative suppression, as can be seen if the data are arranged into three ${2 \times 2}$ contingency tables—one for blood pressure by birth weight, one for blood pressure by current weight, and one for birth weight by current weight. The correlations (${\phi }$ coefficients) computed for each table equal .10, .33, and .38, respectively. The correlation between the explanatory variable (dichotomized birth weight) and the third variable (dichotomized current weight) is greater than the ratio of the correlation of each of these variables with the outcome variable (dichotomized blood pressure): ${.38 > (.10/.33 = .30)}$, indicating that negative suppression has occurred. The regression coefficient for the third variable increases from .33 to .34; the regression coefficient for the explanatory variable reverses sign and decreases in magnitude from .10 to ${-\,.03}$.

### Example of Lord’s Paradox

For their example of Lord’s Paradox, Tu et al. [1] used the continuous outcome variable blood pressure, the dichotomized explanatory (group) variable birth weight, and the continuous third variable current weight. A two-sample *t* test showed that, on average, the blood pressure of persons with a high birth weight is higher than that of persons with a low birth weight. That is, there is a positive and significant association between birth weight and blood pressure. But regressing continuous blood pressure on both dichotomized birth weight and continuous current weight shows a negative association between birth weight and blood pressure. As with their examples of suppression and Simpson’s Paradox, in Tu et al.’s [1] example of Lord’s Paradox, consideration of current weight reverses the association between birth weight and blood pressure, suggesting that low birth weight leads to adult hypertension.

We don’t question the results for this example per se, but this association reversal is not an example of Lord’s Paradox. As explained earlier, Lord’s Paradox compares the effect of the explanatory (group) variable on the outcome variable when the outcome variable is regressed on the explanatory variable and the third variable (residual-score analysis) to the effect of the explanatory (group) variable when the difference between the outcome variable and the third variable is regressed on the explanatory (group) variable (difference-score analysis). There is no such comparison in Tu et al.’s [1] example. Indeed, in this context, such a comparison does not make sense because it would involve the subtraction of current weight from blood pressure. Instead of being an example of Lord’s Paradox, this is an example of negative suppression. Note that the two-sample *t* test comparing blood pressure for persons of low and high birth weight is equivalent to regressing blood pressure on dichotomized birth weight. Thus, Tu et al.’s [1] example compares the results of a regression with one explanatory variable to the results of a regression with two explanatory variables. The latter is the framework for suppression. It does not matter that one of the two explanatory variables is dichotomous; regression can accommodate as explanatory variables dichotomous variables as well as the more usual continuous variables. The correlation between blood pressure and dichotomized birth weight equals .11, the correlation between blood pressure and current weight equals .50, and the correlation between dichotomized birth weight and current weight equals .44. The correlation between the explanatory variable (dichotomized birth weight) and the third variable (continuous current weight) is greater than the ratio of the correlation of each of these variables with the outcome variable (continuous blood pressure): ${.44 > (.11/.50 = .22)}$, resulting in negative suppression and a sign reversal. The regression coefficient for the third variable increases from .50 to .56; the regression coefficient for the explanatory variable reverses sign and increases in magnitude from .11 to ${-\,.13}$ and remains significant.

Tu et al. [1] concluded that consideration of a third variable in epidemiological studies can lead to differences in the strength and the direction of the association between the outcome variable and the explanatory variable of interest and thus affects the interpretation of that association. They correctly indicated that these effects can occur regardless of whether the variables under consideration are continuous, categorical, or some combination of continuous and categorical. They also noted that the question of whether consideration of the third variable yields valid or artifactual results cannot be determined by statistics alone but depends upon prior biological and clinical knowledge and underlying causal theory. In an earlier related article, Tu et al. [22] indicated that they believe that current weight should not be considered in investigations of the association between birth weight and current blood pressure because it is on the casual pathway between birth weight and current blood pressure and so is not a true confounding variable. Consideration of current weight therefore yields results that are statistical artifacts, in their opinion.

## Conclusion

Tu et al. [1] introduced their article by stating that suppression, Simpson’s Paradox, and Lord’s Paradox pervade epidemiological research and have serious implications for the interpretation of evidence from observational studies, and concluded it by noting that these paradoxes cannot be resolved by statistical means. Instead, their resolution requires substantive knowledge, strong theoretical reasoning, and a priori causal models. As psychologists, we are unable to judge whether these paradoxes do in fact pervade epidemiological research. But we agree that they have serious implications for the interpretation of evidence from observational studies, not just in epidemiology, but in all disciplines that employ contingency-table analyses or regression-based models. We applaud Tu et al.’s [1] efforts to bring these paradoxes to the attention of epidemiologists, and heartily agree that their resolution requires more than statistics. But it is also important that the descriptions of the relations between these paradoxes be accurate. To this end, we hope that our corrections and clarifications to Tu et al.’s [1] article prove useful.

## Footnotes

1 Tu et al. [1, p. 7] wrote that “The reversal paradox is often used as the generic name for Simpson’s Paradox, Lord’s Paradox, and suppression (see Table 4). Whilst the original definition and meaning of the reversal paradox was derived from the notion that the direction of a relationship between two variables might be reversed after a third variable is involved, this nevertheless may generalize to scenarios where the relationship between variables is enhanced, not reduced or reversed, after the third variable is introduced.” In our view, this generalization is inappropriate and likely to lead to confusion. Strictly speaking, the reversal paradox requires that a negative relation between two variables become a positive association, or vice versa. A null (zero) association becoming a non-null (positive or negative) association, or vice versa, is not an example of the reversal paradox, although it is an anomaly and a paradox. Reciprocal suppression, in which both partial regression coefficients are larger than their corresponding simple regression coefficients, is not an example of the reversal paradox. Moreover, if the association between an outcome variable and an explanatory variable is reduced for both explanatory variables, this indicates redundancy, not a paradox (reversal or otherwise).

2 To score variables so that the correlation between the two explanatory variables (${r_{X_1X_2}}$) is greater than or equal to zero and the correlation between the outcome variable and the first explanatory variable (${r_{YX_1}}$) is positive may require reversing the original scale of one of the three variables. To reverse the scale of a variable, for each data point compute $\text{reversed value of data point} = \text{maximum value of scale} + \text{minimum value of scale} - \text{original value of data point}$ Reversing the scale of a variable has no effect on the magnitude of its correlation with some other variable; it simply reverses the sign of that correlation.

3 In his example, Simpson [11] coded status (which he called “survival”) $0 = \text{dead}$ and $1 = \text{alive}$, treatment $0 = \text{untreated}$ and $1 = \text{treated}$, and sex $0 = \text{female}$ and $1 = \text{male}$. Thus, in the two subtables for sex (female and male), there was a positive association between treatment and status. For consistency with our presentation of suppression, we have reversed the coding of status and sex. The coding of dichotomous variables is arbitrary; such recoding does not affect the results of the contingency-table analysis.

4 Charig et al. [15] coded kidney-stone size $0 = \text{small}$ and $1 = \text{large}$. For consistency with our presentation of suppression, we have reversed this coding.

5 This data set was found on an Internet website. Unfortunately, we did not record the uniform resource locator (url) for the website and so are unable to credit the person who created the data set.

6 All of the correlations reported in this section were computed from the simulation data set kindly provided to us by Dr. Yu-Kang Tu.

7 Tu et al. [1, Table 2, p. 6] reported the correlation between blood pressure and birth weight to be ${-\,.105}$. The negative sign appears to be a typographical error.
